# Continuation of the genetic divergence of ecological speciation by spatial environmental heterogeneity in island endemic plants

**DOI:** 10.1038/s41598-017-05900-1

**Published:** 2017-07-14

**Authors:** Bing-Hong Huang, Chih-Wei Huang, Chia-Lung Huang, Pei-Chun Liao

**Affiliations:** Department of Life Science, National Taiwan Normal University, Taipei, 11677 Taiwan

## Abstract

Divergent selection plays a critical role not only as a speciation driver but also in maintaining post-speciation divergence. In the absence of direct evidence, ancestral interspecific gene flow between incipient species can reflect ancient selective pressure for ecological speciation. In the present study, two late-Pleistocene diverged species endemic to Taiwan, *Scutellaria playfairii* and *S*. *tashiroi*, were spatially and ecologically partitioned with partial overlap. Multilocus genome-scan analyses and *in silico* evaluation revealed ancestral interspecific gene flow but distinct genetic compositions, implying that adaptive divergence contributed to their speciation. Ecological niche modeling and principal component analysis suggested incomplete divergent niches between the two species; the species distribution is therefore consistent with Hutchinson’s metaphor of multidimensional hypervolume niches rather than attributable to a single factor. Constraint ordination analysis supported this inference of a combination of variables explaining the genetic structure. The rare occurrence of hybrids in the sympatric population suggested hybrid breakdown, providing further evidence of divergent selection blocking gene flow. The correlation of environmental variables with integrated genetic components demonstrated that environmental heterogeneity maintains the species and population differentiation. This study highlights the importance of environmental heterogeneity and divergent selection for the rapid speciation and recent diversification of island plants.

## Introduction

In addition to accelerating speciation, divergent selection is also responsible for maintaining differences between species. Studies have increasingly shown that environmental variables, rather than geographic distance, are responsible for genetic divergence^1–3^, indicating the importance of surrounding environments for species evolution. Via^4^ proposed two stages of selective pressure on ecological speciation and divergence. Stage 1 involves selection against effective migration, and stage 2 involves independent responses to selection within the new lineages. Stage 1 was suggested to clearly define the difference between allopatric and sympatric speciation^4, 5^, whereas directional selection could further independently act on the diverged lineages to reduce the potential for interspecific mating upon physical contact after speciation^4, 6, 7^. However, because past environmental conditions are difficult to record, present environmental heterogeneity can usually explain post-speciation divergence but not the actual driving force for ecological speciation, i.e., only stage 2 of Via can be verified^4^.

Since ecological speciation is a time-dependent process, i.e., genomic barriers for gene flow gradually increase between adaptively divergent species^5, 8^, detecting the degrees of interspecific gene flow through time can support or reject ecological speciation. A selective process leads to species (or population) divergence more rapidly than a stochastic drift process^9, 10^. Hence, gene flow will rapidly be blocked if the selection continues to maintain or enhance the divergence. The development of modeling-based simulation analyses such as the approximate Bayesian computation (ABC) has enabled the evaluation of the best-fit evolutionary scenario of isolation vs. gene flow to resolve the speciation hypothesis. In combination with correlation analysis between genetic variation and environmental variables, the effect of environmental heterogeneity on species differentiation and local adaption can be clarified.

**Figure 1:**
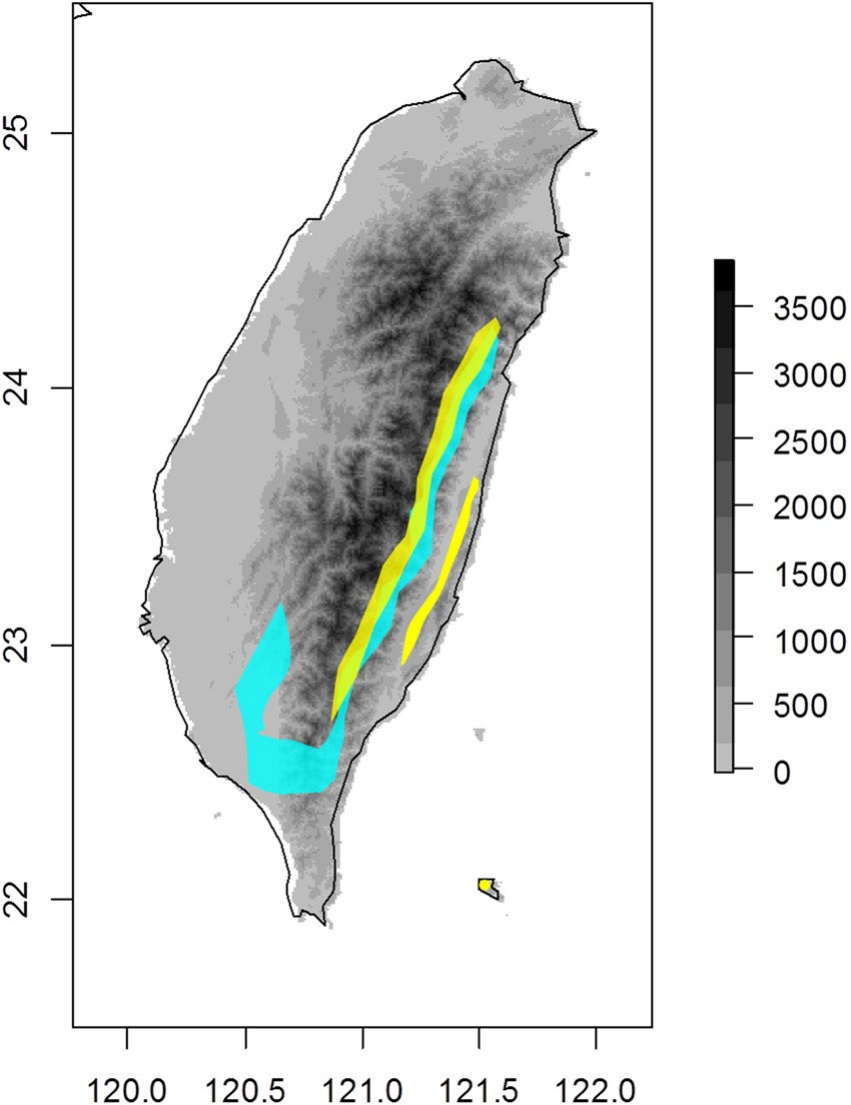
Distribution range of *S*. *playfairii* (light blue) and *S*. *tashiroi* (yellow) in Taiwan. Exact distribution of specimen records is shown in Fig. 5. Despite overlapping distribution ranges in the eastern Taiwan, sympatric populations were found in the southeastern Taiwan only. The map was constructed using the packages maptools^69^ implemented in R.

To investigate the degree to which the genetic diversity may be influenced by the primary speciation process and subsequent environmental pressures, we selected as experimental materials two herb species, *Scutellaria playfairii* Kudo and *S*. *tashiroi* Hayata, which both have a relatively limited spatial distribution. *Scutellaria playfairii* and *S*. *tashiroi* are endemic to Taiwan and are sparsely distributed among low-elevation hills. The *S*. *playfairii* are mainly distributed in the southern Taiwan, while *S*. *tashiroi* are common in the stream valleys of eastern Taiwan. Despite most populations of these two species were allopatrically distributed, there are still some sympatric populations being found in the southeastern Taiwan (Fig. 1). The inflorescence, which can be used to distinguish *Scutellaria* species^11^, is mostly axillary, with rare terminal racemes and dark-purple corollas in *S*. *tashiroi* and terminal, loose racemes and whitish-purple corollas in *S*. *playfairii* (Supplementary Table S1). Transcriptomic-based analysis has shown that the growth form and flower color-related transcription factors (MYBs) are positively selected and are possibly related to the differentiation of flower color and speciation^12^. Divergent selection and functional subdivision of anthocyanin-biosynthetic genes have also been suggested to be responsible for the rapid evolution and diversification of *Scutellaria* species in the island of Taiwan^13, 14^. In addition, biogeographic analyses have indicated a very recent divergence time, within 0.2 Mya, of these two species, and their divergence was suggested to be a consequence of a series of dispersal and vicariance events corresponding to climate shifts through the late Pleistocene^15^. Natural selection and biogeographic expansion may together explain the rapid diversification of these two island species.

Because geo-environmental heterogeneity can act as a selective agent for species and population differentiation, ecologically divergent selection and local adaptation may be valid explanations of species differentiation and population structure. Quantifying the effects of environmental factors on geographic and genetic distributions can aid the clarification of the mechanisms driving and maintaining species/population differentiation. For example, multivariate analyses that using linear dependent similarity matrix can assess how does the constraint factors (e.g. environmental or/and geographic distances) affect the response (e.g. genetic distance)^16, 17^. Furthermore, modeling species distribution using climatic variables (ecological niche modeling, ENM) can be used for exploring the ecological association and discriminating environmental requirements of each species, which are widely used in testing hypotheses of ecological divergence and speciation^18, 19^. The hypothetico-deductive evolutionary analyses, such as the ABC approach, provide powerful statistical verification method to evaluate the best speciation model by using summary statistics and simulation without exact likelihood calculations^20, 21^. The ABC is widely used in unravelling complex evolutionary history such as the relict cycad species^22^, phylogenetic closely related skullcap flowers^23^, *Dysosma versipellis-pleiantha* complex^24^, etc. Through integration of these frameworks, we can test the ecological divergence of *S*. *tashiroi* and *S*. *playfairii*, and evaluate the relative importance of each environmental factor on their genetic divergence. These integrated approaches has successfully applied in another two Taiwanese *Scutellaria* species to confirm their taxonomic relationship^23^.

In this paper, we assessed the ecological speciation and post-speciation adaptive divergence of these two recently diverged *Scutellaria* species through *in silico* speciation-processing modeling and correlations between climatic variables and genetics. Here, we extract environmental factors that cause or sustain genetic differences and highlight the important role of integrated environmental elements in plant genetic make-up.

## Results

### Genetic variation and population structure

According to the genetic assessment of 19 microsatellite loci, *S*. *playfairii* and *S*. *tashiroi* showed similar genetic diversity with respect to expected heterozygosity (ranges: 0.231 ± 0.061~0.340 ± 0.067 in *S*. *playfairii* and 0.253 ± 0.069~0.371 ± 0.069 in *S*. *tashiroi*) and the inbreeding coefficient (fixation index: 0.431 ± 0.085~0.727 ± 0.053 in *S*. *playfairii* and 0.387 ± 0.061~0.663 ± 0.043 in *S*. *tashiroi*) (Table 1). The moderate heterozygosity levels and inbreeding coefficients suggest self-compatibility in both species, consistent with the inference of a selfing or sibling-mating tendency in small and isolated populations of *S*. *montana* ^25^ and *S*. *indica* ^26^. The BayeScan approach found three positive outlier loci and one negative outlier locus (Supplementary Fig. S1), which were suggested to be candidate loci under positive selection and balancing selection, respectively^27^. Analysis of molecular variance (AMOVA) based on the remaining 15 neutral loci revealed a non-significant difference in genetic variation between species but a significant divergence between populations (Table 2). Less than half of the total genetic variation was due to between-species differences in total loci and neutral loci (47.30% and 39.88%, respectively), whereas 90.51% of the genetic variation was due to between-species differences in adaptive loci, with a smaller contribution from population effects (Table 2). However, non-significant genetic differences were detected between species in all three datasets (*P* = 0.107, 0.093, and 0.096, respectively, Table 2). By contrast, significant genetic differences were detected between populations within species, implying adaptive divergence among populations (local adaptation) for both plant species.

**Table 1:** Genetic diversity of each sampled population as inferred by 19 microsatellite loci.

	*S. playfairii*				*S. tashiroi*					
	Wutai		Wulu		Wulu		Lanyu		Taroko	
	Mean	SE	Mean	SE	Mean	SE	Mean	SE	Mean	SE
*N*	10		19		22		15		15	
*Na*	2.526	0.319	2.000	0.276	3.158	0.735	2.158	0.428	2.632	0.466
*Ne*	1.865	0.201	1.553	0.193	1.924	0.328	1.737	0.276	1.882	0.252
*I*	0.605	0.122	0.396	0.107	0.543	0.167	0.447	0.132	0.583	0.136
*Ho*	0.179	0.047	0.066	0.026	0.098	0.035	0.126	0.057	0.204	0.053
*He*	0.340	0.067	0.231	0.061	0.259	0.078	0.253	0.069	0.317	0.069
*uHe*	0.358	0.071	0.237	0.062	0.265	0.079	0.262	0.071	0.328	0.072
*F*	0.431	0.085	0.727	0.053	0.663	0.043	0.599	0.089	0.387	0.061

*N*, sample size; *Na*, number of different alleles; *Ne*, number of effective alleles; *I*, Shannon’s information index; *Ho*, observed heterozygosity; *He*, expected heterozygosity; *uHe*, unbiased expected heterozygosity, correcting the *He* using the formula (2 *N*/(2*N* − 1)) × *He*; *F*, fixation index.

**Table 2:** Summary table for the analysis of molecular variance (AMOVA).

	Source of variation	df	SS	Var Comp.	%Var	*F* statistic	*P*
Total loci	Among species	1	323.240	3.669	47.30	0.473	0.107
	Among populations within species	3	142.135	1.423	18.34	0.348	0
	Within populations	157	418.576	2.666	34.37	0.656	0
	Total	161	883.951	7.758			
Neutral loci	Among species	1	215.730	2.331	39.88	0.399	0.093
	Among populations within species	3	119.857	1.198	20.49	0.341	0
	Within populations	157	363.598	2.316	39.63	0.604	0
	Total	161	699.185	5.844			
Positive outlier loci	Among species	1	103.292	1.325	90.51	0.905	0.096
	Among populations within species	3	13.103	0.139	9.49	1	0
	Within populations	157	0	0	0	1	0
	Total	161	116.395	1.464			

**Figure 2:**
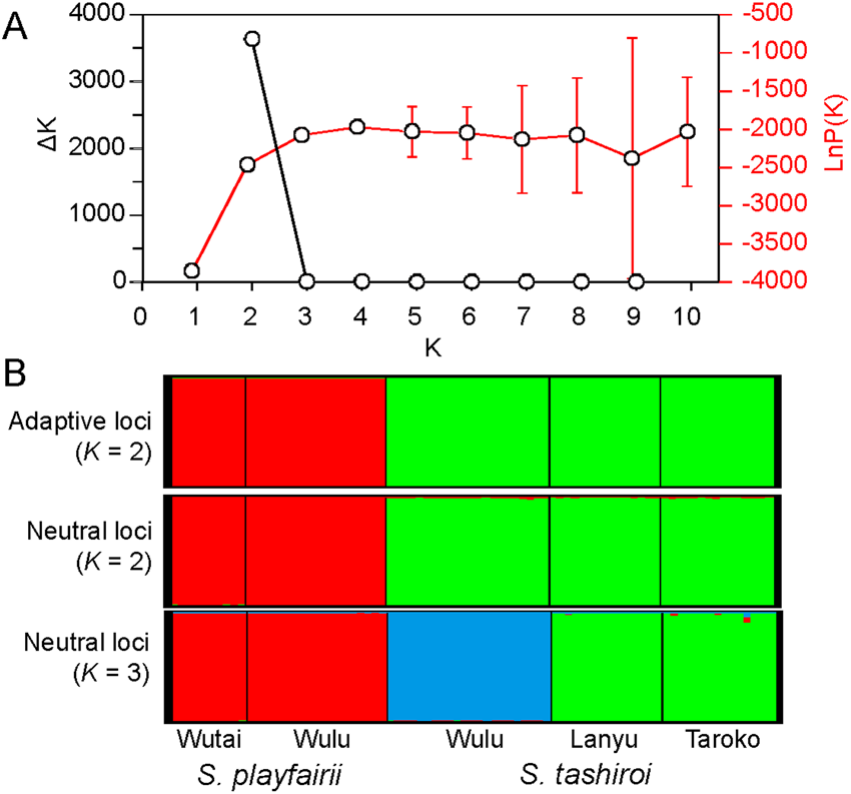
Results of Bayesian clustering analysis conducted by STRUCTURE. (**A**) The changes in Δ*K* and lnP(*K*) in different clustering (*K*) situations in neutral loci. The Δ*K* plot shows that the highest Δ*K* value occurs at *K* = 2. This plot also shows lnP(*K*), which demonstrates the increase in the posterior probability of *K*. (**B**) The clustering patterns of genetic components by two groups (*K* = 2) in adaptive loci (positive outliers) and the patterns by two (*K* = 2) and three groups (*K* = 3) in neutral loci.

**Figure 3:**
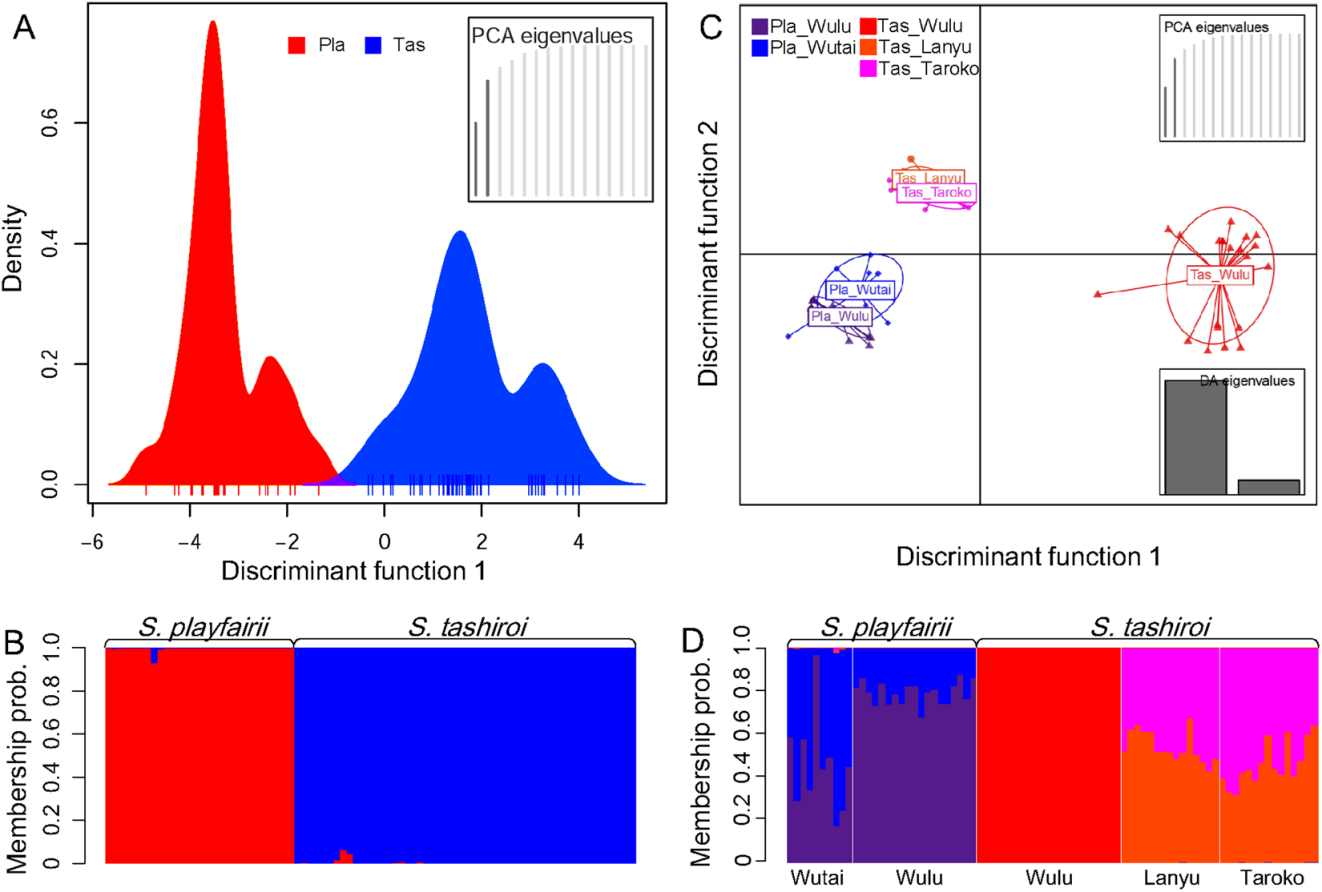
Genetic structure of the sampled populations as estimated by discriminant analysis of principal components (DAPC). The results show discriminable groups at the species level in the scatter plot (**A**), with small fractions of genetic admixture in the compoplot (**B**), and clusters of populations with partial overlap (**C**) and obvious admixture patterns between populations of each species, except the Wulu population of *S*. *playfairii* (**D**).

We then performed Bayesian clustering analysis (BCA) and discriminant analysis of principal components (DAPC) to illustrate the grouping patterns of these five populations. BCA suggested that the best clustering number was two (*K* = 2), according to the highest Δ*K* (lnP(*K*) = −2465.31, ln’(*K*) = 1393.59, |ln”(*K*)| = 1005.47, Δ*K* = 3633.97, Fig. 2A), corresponding to groups of species in both the adaptive- and neutral-locus datasets (Fig. 2B). In the neutral-locus dataset, *K* = 3 was also optimal, separating the Wulu population of *S*. *tashiroi* from the other two *S*. *tashiroi* populations (Fig. 2B), a pattern similar to that of the DAPC for neutral loci. DAPC showed clear differentiation between the species when only the first two PCs were considered (Fig. 3A,B). At the population level, genetic admixture was observed between the two populations of *S*. *playfairii*, whereas the Wulu population of *S*. *tashiroi* was distinguished from the other two populations (Fig. 2C,D). The DAPC scatterplot revealed similar genetic compositions of the Lanyu and Taroko populations of *S*. *tashiroi* (Fig. 3C) due to similar admixture patterns of genetic components (Fig. 3D). It is worth to notice that the Wulu population of *S*. *tashiroi* has distinguished but non-admixed genetic component from other populations of both species under *K* = 3 in BCA and DAPC (Figs 2B and 3D). Genotypes of *S*. *tashiroi* in Wulu neither exhibit intermediate form (i.e. heterozygotes or chimera) between any populations of two species, means that the distinguished genetic cluster of the Wulu population of *S*. *tashiroi* is not attributed to hybridization between two species.

All three positive outlier loci were homozygous (Supplementary Table S2); however, loci aus9-2 and aus9-5 are species-private alleles. The other locus, aus9-3, has three fixed genotypes corresponding to the Wulu and Wutai populations of *S*. *playfairii* and the species *S*. *tashiroi*. These results suggest an adaptive divergence of the two species and local adaptation among populations of *S*. *playfairii*. DAPC analysis could not be performed using positive outlier loci due to the homozygosity of all samples. Incongruent grouping by neutral (*S*. *playfairii*; Wulu of *S*. *tashiroi*; Taroko and Lanyu of *S*. *tashiroi*) and adaptive loci (Wulu of *S*. *playfairii*; Wutai of *S*. *playfairii*; *S*. *tashiroi*) suggested that drift (neutral) processes and selective pressures affected population genetic structure and species divergence simultaneously but not synchronously.

### Speciation model

**Figure 4:**
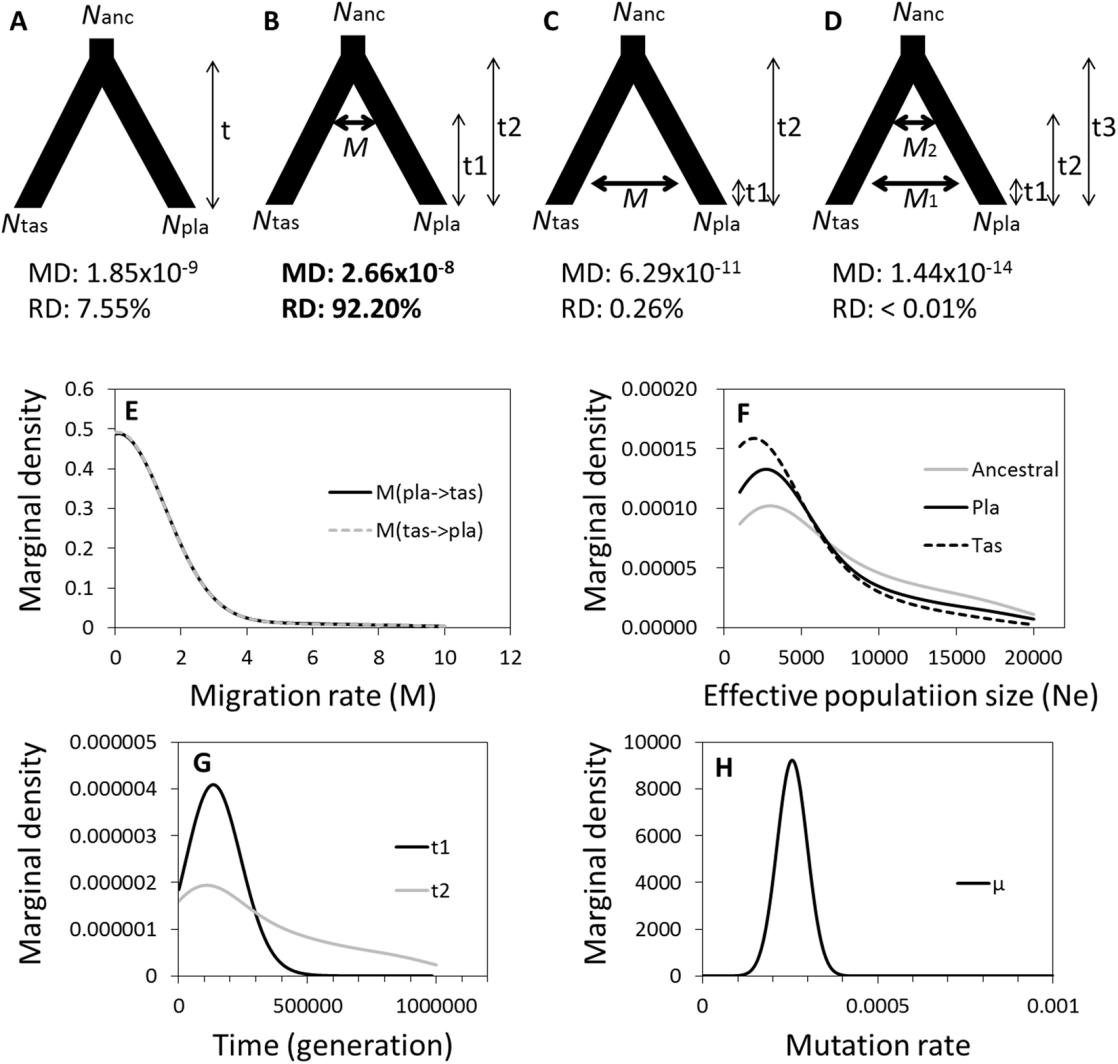
Evolutionary scenarios of speciation between the phylogenetically close species *S*. *tashiroi* (tas) and *S*. *playfairii* (pla) and the estimated consequences of approximate Bayesian computation: (**A**) the CI model; (**B**) the AM model; (**C**) the SC model; and (**D**) the CM model. The AM scenario had the highest posterior probability. Distributions of the estimated parameters for the best scenario (AM) are presented: (**E**) migration rate, (**F**) effective population sizes, (**G**) divergent time and the time of gene flow, and (**H**) average mutation rate of 15 neutral microsatellite loci. MD, marginal density; RD, relative density.

An ABC framework permitted an evaluation of speciation scenarios for *S*. *playfairii* and *S*. *tashiroi*. Among four speciation scenarios (see Methods), the ancestral migration (AM) model (marginal density: 2.66 × 10^−8^, relative density: 92.20%) describing early interspecific gene flow after divergence best explained the current genetic divergence of *S*. *tashiroi* and *S*. *playfairii* relative to the complete isolation (CI), secondary contact (SC), and continuous migration (CM) models (Fig. 4). We detected equal rates of bidirectional gene flow (Mode of *M*_tas→pla_ = *M*_pla→tas_ = 0.100, i.e., 10% of individuals are migrants per generation, Supplementary Table S3) at the beginning of speciation under the AM model. The estimated time of gene flow (*t*_1 mode_ = 134474 generations, *t*_1 median_ = 150300 generations) was similar to the divergence time (*t*_2 mode_ = 111448 generations, *t*_2 median_ = 280534 generations). *Scutellaria* species are annual herbs. Consequently, the divergence and early interspecific gene flow can be traced back to the early Pleistocene. This estimate of the divergence time is thus consistent with the estimate of <0.2 Mya based on nuclear and chloroplast genes^15^. The detailed parameter estimates of the AM model are listed in Supplementary Table S3.

### Niche differentiation between *S. playfairii* and *S. tashiroi*

After removing variables of multicollinearity, the remaining five environmental variables with the smallest variance inflation factor [VIF, including altitude (alt), mean actual annual evapotranspiration (AET), isothermality (bio3) and precipitation in the wettest (bio13) and driest months (bio14)] revealed partial overlap of niches between the two species in principal component analysis (PCA) space (Supplementary Fig. S2). The first three PCs explained 44.20%, 33.68%, and 10.87% of the environmental variance. The Kruskal-Wallis rank sum (KW) test was further performed to test significant differences between the species. Only PC1 revealed significant species differentiation (KW test: *χ* ^2^ = 7.839, df = 1, *P* = 0.0051, Supplementary Fig. S3), whereas the other two PCs did not indicate significant differences between the species (PC2: KW test: *χ* ^2^ = 0.833, df = 1, *P* = 0.3614; PC3: KW test: *χ* ^2^ = 0.364, df = 1, *P* = 0.5463, Supplementary Fig. S3). The general linear model (GLM) further revealed that all five environmental variables were significantly correlated with the species-divergent PC1 (Supplementary Table S4). These correlations indicate that these five variables are all responsible for the explanation of niche divergence on PC1. We next examined whether these environmental parameters explain the niche differentiation between species together or independently by performing multivariate logistic regression to assess the relationships between the dependent variables (i.e., species) and each independent variable (i.e., environmental variables or the PCs), controlling for the other independent variables. None of the environmental variables directly predicted species differentiation alone, but the reduction axis PC1 of these environmental variables had a significant effect on predicting species divergence (*Z* = 2.626, *P* = 0.009, Table 3). This result suggests that the niche differentiation between species cannot not be explained by any single environmental factor but rather by the contributions of a variety of environmental factors.

**Table 3:** Multivariate logistic regression to test the effect of environmental variables on species differentiation.

	Coefficient	SE	*Z*	*P*
Formula: species~alt + AET + bio3 + bio13 + bio14				
Intercept	5.362	24.257	0.221	0.825
alt	−0.004	0.004	−1.123	0.261
AET	−0.017	0.023	−0.724	0.469
bio3	0.400	0.303	1.321	0.187
bio13	−0.013	0.009	−1.425	0.154
bio14	0.056	0.040	1.39	0.165
Formula: species~PC1 + PC2 + PC3				
Intercept	−0.495	0.425	−1.165	0.244
PC1	0.962	0.366	2.626	0.009*
PC2	−0.480	0.379	−1.264	0.206
PC3	−0.959	0.781	−1.228	0.220

*P < 0.01.

### Ecological niche modeling

**Figure 5:**
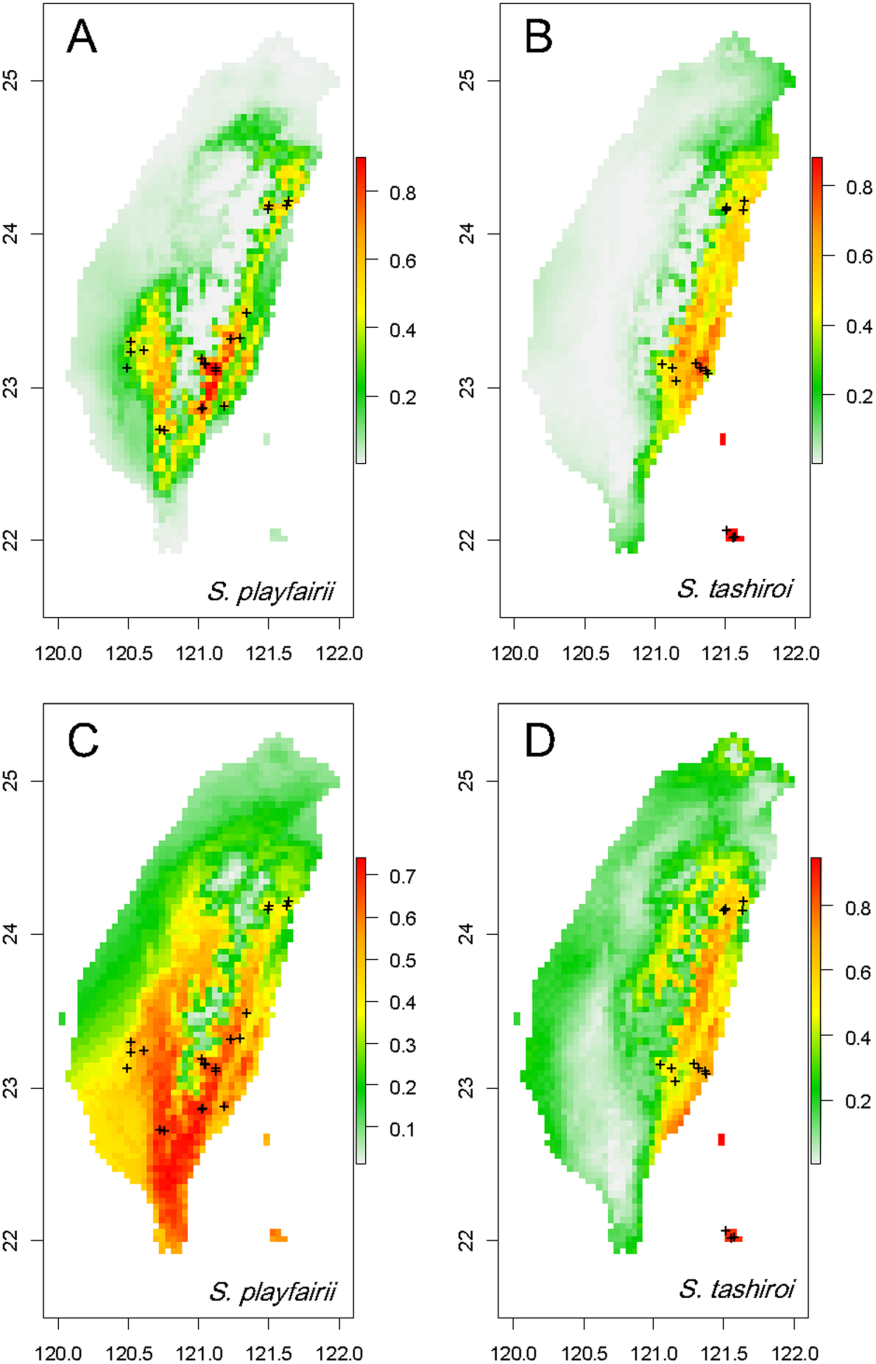
Ecological niche modeling for predicting the potential distributions of *S*. *playfairii* and *S*. *tashiroi*. (**A**,**B**) are the potential distributions inferred by all 71 collected environmental variables; (**C**,**D**) were predicted by the remaining five variables after removing variables of multicollinearity. The map was constructed using the packages maptools^69^ implemented in R.

To model the niches of *S*. *playfairii* and *S*. *tashiroi* and compare ecological associations between these two phylogenetically close species, the potential species distribution was modeled on a map of Taiwan according to total 71 environmental variables (ENM1, Supplementary Data) or five remaining variables without multicollinearity (ENM2). The average test of the cross-validated area under the curve (AUC) for predicting the distribution of *S*. *playfairii* was 0.830 (±0.171) and 0.841 (±0.093) for replicate runs of ENM1 and ENM2, respectively; the AUC for predicting the distribution of *S*. *tashiroi* was 0.921 (±0.025) and 0.888 (±0.047) in ENM1 and ENM2, respectively. The high AUC value indicates good performance in modeling the distributions of both *Scutellaria* species. The range of species distribution predicted by ENM1 is closer to the actual recorded sites of *S*. *playfairii*, with a likely distribution in low-altitude mountains of eastern Taiwan and lowlands of southwestern Taiwan (Fig. 5A), whereas the likely distribution of *S*. *tashiroi* is in the lowlands of eastern Taiwan and islets off southeastern Taiwan (Fig. 5B). When the predicting variables were reduced to five (ENM2), the potential distribution of *S*. *playfairii* expanded to the south and included more lowland areas of southeastern and southwestern Taiwan (Fig. 5C). By contrast, the potential distributions of *S*. *tashiroi* predicted by ENM1 and ENM2 were similar (Fig. 5D). ENM1, which used all available predictors, provided a better fit to the real distributions than ENM2, indicating that the present distributions of the extant populations of these two species might be sensitively impacted by local- or micro-environmental variation.

### Explanation of the role of environmental variables in plant genetic diversity

According to dbRDA, environmental variables explained 90.83% of the genetic variation of the two species (Supplementary Table S5), and four constraint variables (alt, AET, bio3, and bio14) significantly influenced the genetic structure of the plant populations (type II analysis of variance (ANOVA), *P* < 0.001, Supplementary Table S6). By contrast, bio13 did not significantly explain the genetic variation in all repeated analyses in which any other variable was removed (not shown). The distribution of these four significantly explanatory variables along the ordination axes was further examined by GLM, which revealed a significant correlation (*P* < 0.05) of all variables along axis 1 and axis 2, except a marginal significance in bio3 along axis 1 (*P* = 0.0717, Table 4). Four environmental variables had significant *F* statistics (adjusted *R* ^2^ = 0.704, 0.475, 0.362 and 0.749 for alt, AET, bio3, and bio14, respectively, Table 4). The high adjusted *R* ^2^ indicates that these estimates sufficiently explain these two axes of dbRDA. Consistent estimates of influence were obtained even when the order of predictors was altered; thus, these four variables are highly relevant for explaining the genetic structure of the plant populations.

**Table 4:** The general linear model (GLM) for testing the correlation of the constraint variables with the first two ordination axes of dbRDA.

	Axis 1				Axis 2				Adj *R* ^2^	*F*	*P*
	Estimate	SE	*t*	Pr(>|*t|*)	Estimate	SE	*t*	Pr(>|*t*|)			
alt	1.409	0.237	5.941	7.49E-08	2.698	0.216	12.489	<2.00E-16	0.704	96.07	<2.00E-16
AET	−0.400	0.118	−3.400	0.0011	−0.847	0.107	−7.906	1.43E-11	0.475	37.2	4.53E-12
bio3	0.009	0.005	1.826	0.0717	0.031	0.005	6.627	4.00E-09	0.362	23.7	9.09E-09
bio14	−0.115	0.029	−3.929	0.0002	−0.399	0.027	−15.001	<2.00E-16	0.749	120.6	<2.00E-16

The distributions of the sampled populations in the space of constraint ordination analysis by dbRDA were similar to the DAPC clustering patterns (Fig. 3C,D). The scatter and ordisurf plots for each environmental variable congruently showed broader ranges of environmental contours in populations of *S*. *tashiroi* compared with populations of *S*. *playfairii* (Supplementary Fig. S4). The Wulu population of *S*. *tashiroi* exhibited a different distribution of the space of dbRDA along axis 1 but similar contour ranges of environmental variables compared with the populations of *S*. *playfairii*, reflecting its sympatric distribution with *S*. *playfairii* in Wulu. By contrast, the distributions of the allopatric Lanyu and Taroko populations of *S*. *tashiroi* were too close to be distinguished in the dbRDA space and were positively associated with the explanatory elements AET and bio14 (Supplementary Fig. S4). Ordisurf plots clearly illustrated the niche differentiation between *S*. *playfairii* and *S*. *tashiroi*. However, it must be noted that this conclusion is only based on the results of estimation from a small number of populations (two populations of *S*. *playfairii* and three populations of *S*. *tashiroi*).

## Discussion

### Genetic evidence of non-allopatric speciation

This study provides a comprehensive assessment of the role of evolutionary background and environmental effects on the current genetic distribution of plant populations. The state-of-the-art ABC method was used to investigate the process of reproductive isolation (RI) (i.e., barriers to gene flow) between *S*. *playfairii* and *S*. *tashiroi*. Interspecific gene flow was detected at the beginning of speciation (Fig. 4), implying that divergent pressure occurred gradually rather than instantaneously^28^. In this ancestral gene-flow mode of speciation, divergent ecological adaptation may play a critical role in driving and maintaining RI^29^. However, the divergence of *S*. *playfairii* and *S*. *tashiroi*, which have very small estimated bidirectional migration rates and overlapping time estimates of divergence and gene flow (Fig. 4 and Supplementary Table S3), could be driven by integrated mechanisms, including adaptation and demographic change, e.g., vicariance^15^. In other words, adaptive and stochastic (drift) processes are not mutually exclusive for explaining the speciation of these two *Scutellaria* species. Although *S*. *playfairii* and *S*. *tashiroi* recently diverged (roughly 10^5^ generations ago, Fig. 4 and Supplementary Table S3), the differentiated habitat preference likely increased the successful RI between these species. Such niche differentiation is attributable not to a single environmental factor but to the overall environmental heterogeneity (Table 3), consistent with Hutchinson’s metaphor of a multidimensional hypervolume niche^30^. However, the environmental variables used for niche analysis were present-day data rather than from ancient time; thus, the factors that we detected as responsible for species divergence are not the driving force for speciation but are likely mechanisms for maintaining or even for secondary accumulation of genetic divergence after speciation^31^.

The distribution of plants has been suggested to be synchronously affected by postglacial colonization and environmental heterogeneity^32, 33^. Drastic climatic change in the late Pleistocene may have resulted in distributional divergence between *S*. *playfairii* and *S*. *tashiroi*, which diverged roughly 0.2 Mya (as estimated by nuclear and chloroplast DNA sequences^15^) or <0.15 Mya (as estimated by microsatellite markers in this study). Larger-scale studies have demonstrated that both climate and geo-historical factors can explain the plant diversity of eastern Asian archipelagos, reflecting as well the interaction of recent ecological and evolutionary processes^34–36^. The inference of late Pleistocene divergence with initial gene flow (Fig. 4) is congruent with the inference of climate-dependent dispersal/vicariance events by Chiang *et al*.^15^. Furthermore, *Scutellaria* exhibit passive seed dispersal by water^37^ as well as high selfing or inbreeding rates due to the occasional cleistogamous flower^26, 38^. Such dispersal and mating mechanisms limit gene flow and favor divergence between populations (Table 2). The characteristic of self-compatibility also reduces the dilemma of fitness reduction of incipient species due to loss of pollinators ^cf.^ ^39^.

### Ecological divergence maintains post-speciation reproductive isolation

After speciation, cyclic glacial/interglacial epochs influenced the population genetic structure of *Scutellaria* species in Taiwan^15^. The late Pleistocene climate cannot be obtained directly, but our results indicate a significant role of current environmental heterogeneity in shaping the genetic structure of *Scutellaria* populations (Table 4 and Supplementary Table S6). Several studies have also inferred that the spatial heterogeneity of local climates is responsible for genetic structure and demography in a variety of organisms^40–43^. Recent studies have also suggested that several environmental variables are together involved in RI between species^44, 45^ or in the genetic structure within species in Taiwan^46^.

Micro-environmental differences reduce the chance of population inter-diffusion and prevent genetic secondary contact if physical contact occurs^47^. An example is the sympatric populations of both species in Wulu, where both species bear similar environmental pressure (e.g., temperature and precipitation), with slight differences in micro-environments (e.g., loam substrate and more humid in the microhabitat of *S*. *playfairii*; sand substrate and more arid in the microhabitat of *S*. *tashiroi*, Supplementary Table S1 and Supplementary Data). In our field observations, a few hybrids were found but were not widely spread in the distribution range of Wulu, suggesting that the fitness reduction of hybrids reduced the intensity of hybrid expansion (i.e., extrinsic postzygotic isolation or hybrid breakdown). Although we cannot completely rule out the probability that the unique genetic cluster of Wulu population in *S*. *tashiroi* in BCA (*K* = 3, Fig. 2B) is a consequence of asymmetric gene flow from the sympatric population of *S*. *playfairii*, the low observed heterozygosity (0.098), no chimera individuals, and lower marginal density of recent contact model (SC and CM, Fig. 4) in ABC together reduce the possibility of unequal rates of introgression leading unique genetic cluster in Wulu population of *S*. *tashiroi*. The better explanation for the unique genotype of Wulu should be caused by a lack of effective gene flow with other *S*. *tashiroi* populations over a long term, rather than the acquisition of genes from other species. These observations support our speculation that the ecological heterogeneity between these two species plays a critical role in maintaining species divergence.

Notably, the integrated variable (i.e., PC1, Table 3) but no single environmental variable differentiated the species, implying that the observed niche differentiation can be explained as a consequence of adaptive divergence instead of as a driving force for speciation. Adaptive divergence for speciation is usually initiated by single or simple environmental differences, followed by the expansion of the accumulating genetic divergence to the whole genome and subsequent niche divergence^5, 48^. This integration of environmental factors in the niche-allocation explanation is also reflected in the results of the more accurate spatial distribution prediction using multiple environmental predictors (Fig. 5). In this study, we used neutral markers to represent the genetic background, and hence the estimated population/species divergence is a surrogate for the degree of RI ^cf.^ ^47^. The significant correlation between the environmental variables and neutral genetic diversity suggests that the demographic dynamics and population structure are governed by environmental variation (Table 4 and Supplementary Tables S5 and S6). The lack of correlation among genetic, geographic, and environmental distances (Supplementary Note online) also implies that the genetic structure of the current population cannot be explained only by the current geographic distribution and environments but should also be jointly explained by the evolutionary history (paleodistribution, ancient selective pressures, etc.).

In our sampled population, the nonsignificant genetic differentiation between species estimated by AMOVA (Table 2) is contrary to the population structure estimation by BCA (Fig. 2) and DAPC (Fig. 3). This discrepancy is likely attributable to masking of the genetic delimitation of species in AMOVA by the high proportion of common ancestral polymorphisms due to recent divergence. However, the significant population differentiation estimated by AMOVA of either neutral loci or positive-outlier loci (Table 2) supports the inference that local adaptive divergence has already facilitated population differentiation. Traditionally, the genetic diversity of neutral genes is expected to be correlated with geographic distance and demographic events, whereas local environmental pressures act on certain “adaptive” genes under natural selection^49^. However, the lower fitness of immigrants could increase the genetic divergence between nearby populations and further result in correlations of neutral genetic variation with local environmental heterogeneity by facilitating genetic drift ^cf.^ ^47, 50, 51^. In other words, our data suggest that local environmental heterogeneity extends the ecological divergence among populations, i.e., post-speciation adaptive divergence.

## Conclusions

Adaptation divergence is usually involved in speciation on islands with rugged topography and varied environments. It is difficult to detect the source of natural selection that leads to species divergence at the initial stage of speciation, but adaptive divergence can be genetically detected via heterogeneous genomic differentiation^52^ and ancestral gene flow between species^5^. In addition, environmental pressures for maintaining and enhancing species divergence, even local adaption between populations, can be tested by correlation analysis of genetic variation and environmental variables^47^. Here, we provided a case of ecological speciation and local adaptive divergence between two island herb species with limited distributions and clarified the mechanisms influencing the population genetics of these plants. We inferred that the evolutionary history, i.e., late Pleistocene divergence marked by early interspecies gene flow, and the incorporation of climate pressures shaped the current genetic distribution of *Scutellaria* species in eastern Taiwan. Integrated environmental factors jointly maintained the species divergence and shaped the current genetic spatial distribution. Our study provides a comprehensive assessment of the historical and environmental effects impacting the local adaptation of insular plants.

## Materials and Methods

### Study sites for genetic analyses

Two populations of *S*. *playfairii* (Wutai and Wulu) and three populations of *S*. *tashiroi* (Taroko, Wulu, and Lanyu) were sampled for genetic accession. These populations all differed in terms of habitat characteristics (e.g., soil types, shade, and precipitation; Supplementary Table S1). Wulu is the only location in which both species are sympatrically distributed. Lanyu is a small island off southeastern Taiwan. Hence, our study system includes two different plant species with both allopatric and sympatric distributions, enabling an understanding of how different environmental conditions affect the genetic divergence of plants.

### Genetic analyses

The evaluation of the genetic diversity of plants was based on 19 selected microsatellite loci, adapted from Chiang *et al*.^53^ (Supplementary Data). Neutral loci and candidate adaptive loci (positive outliers) were determined based on a multinomial-Dirichlet distribution under simulations of reversible-jumped Markov chain Monte Carlo (rjMCMC) using BayeScan 2.1^54^. Outlier loci were determined after running ×10^5^ iterations with a thinning interval of 1000, posterior odds (PO) >10 (strong selection *P* = 0.90), and a *q*-value < 0.05 for the false-discovery rate as the threshold settings. We conducted DAPC and BCA using the function dapc in the R^55^ package adegenet^56^, and we used STRUCTURE 2.3.4^57^ to discriminate genetic clusters. DAPC is a “without *a priori*” method using partial synthetic variables to minimize variation within groups^58, 59^, whereas BCA is a population-model-based approach based on the Hardy-Weinberg and linkage equilibria^60^. For DAPC, groups of “species” and “populations” were set to conduct the DAPC separately, and the first two principal components (PCs) were selected to discriminate groups. For BCA, the individual assignment was conducted under admixture model without incorporating locality information (i.e. without LOC prior). The correlated allele frequency model was assumed for BCA. Total 10 independent runs were conducted for BCA. Each run contains 1 million MCMC simulation followed by 100 thousand steps burn-in. These 10 runs were aligned and summarized using CLUMPP^61^. Analysis of molecular variance (AMOVA) by hierarchical groupings of species and populations permitted an assessment of population structure.

### Evaluating speciation models by approximate Bayesian computation

We tested four scenarios by ABC: (1) CI model: gene flow was not permitted during divergence (Fig. 4A); (2) AM model: gene flow persisted only during initial divergence (Fig. 4B); (3) SC model: gene flow occurred in late periods of divergence (Fig. 4C); (4) CM model: gene flow persisted continuously during divergence (Fig. 4D). We set the migration rate as a free parameter in both directions in AM, SC, and CM models, which let both situations of symmetric and asymmetric interspecific gene flow (introgression) to be considered in each migration model. For performing the ABC, we used 15 neutral microsatellite loci and discarded four outlier loci for analysis, because these outlier loci could be adaptive under positive or balancing selection^62^ and could exhibit different evolutionary scenarios to neutral loci^52, 63, 64^. One million simulations were generated using the package simcoal2 implemented in ABCtoolbox^65^. The summary statistics were reduced to five partial least-squares components following the instructions in ABCtoolbox. Using the R package ABCestimator, we performed a general linear model post-sampling regression adjustment on the best 5,000 simulations to obtain the marginal density of each model. We selected the best model scenario using the Bayes factor and ratio of marginal densities for each model.

### Niche differentiation between species estimated by reduction of environmental factors

To infer the niche distributions of *S*. *playfairii* and *S*. *tashiroi*, a total of 71 climatic variables were retrieved from WorldClim [elevation (alt), monthly precipitation (prec), temperature (tmin and tmax), water vapor pressure (vapr) and bioclim, http://www.worldclim.org/bioclim] and Consultative Group for International Agricultural Research - Consortium for Spatial Information (CGIAR-CSI) [global aridity, potential evapotranspiration (PET), and actual evapotranspiration (AET), http://www.cgiar-csi.org/data] (Supplementary Data). The occurrence data of both species were obtained from our sampling sites, herbarium records, and Global Biodiversity Information Facility (GBIF, http://www.gbif.org). Total 75 (*S*. *playfairii*) and 78 (*S*. *tashiroi*) records have been obtained. Species occurrence data were checked and cleaned. We followed instruction in dismo packages implemented in R^66^. Repeated records (multiple records of the same species in the same grid) of occurrence sites were removed to leave one record only in order to avoid unnecessary weight. Furthermore, each specimen was checked if available, and occurrence records of misidentified species were discarded. Occurrence data that did not project onto land were removed. Finally, twenty-three and sixteen records were preserved for *S*. *playfairii* and *S*. *tashiroi*, respectively. Variables with a high VIF were removed to reduce multicollinearity. We repeated this procedure until the VIF values of all remaining variables were <10, and finally, five environmental variables, alt, AET, bio3, bio13, and bio14, were retained for further analyses.

First, PCA was conducted to reduce the dimensionality of these five variables defining the niche space, thus enabling comparison of the integrity of Grinnellian niches between *S*. *playfairii* and *S*. *tashiroi*. Next, we performed the Kruskal-Wallis rank-sum test to detect differences in environmental variables between species. Then, we analyzed the distribution of environmental variables along the PCs of significant species differences by GLM to confirm the significance of the correlation of variables on the PCs. Multivariate logistic regression was further conducted to test whether the environmental variables significantly predicted species differentiation.

### Ecological niche modeling to predict the species distribution pattern

To demonstrate the spatial distributions of the environmental niches of the two species, ecological niche modeling (ENM, also known as species distribution modeling) was performed under the maximum entropy model implemented in MaxEnt^67^ and the R package raster^68^. For ENM, 71 environmental variables (ENM1) and five retaining variables (ENM2) were used to predict the potential species distributions. All variables were re-scaled to a resolution of 30 arc-sec (approximately 1 km × 1 km). A maximum of 2000 iterations was conducted for each species, and the species occurrence data were randomly divided (10%) to train the model. One regularization multiplier and 10,000 background points were set to create models for each species set. The logistic output consisting of a grid map with a probability value ranging from 0 to 1 was generated and visualized with the R package maptools^69^. The predicted models were evaluated by the AUC value, which is a ranked approach for evaluating model fit and determines the probability that a presence location is ranked higher than a random simulation.

### Redundancy analyses of environmental variables for assessing influence on genetic variation

To test environmental effects on the genetic variation of the sampled populations of *S*. *tashiroi* and *S*. *playfairii*, dbRDA was conducted with the first two principal components (PC1 and PC2) of the microsatellite data as the genetic response and the remaining five environmental variables (alt, AET, bio3, bio13, and bio14) after VIF analysis as the environmental predictors. However, since only five populations were sampled for genotyping, which limits the number of variables applying to the genetic response, we repeated the dbRDA several times by discarding one of the environmental predictors. Finally, we found that bio13 could not obtain a significant explanation in any analysis. Thus, bio13 was excluded, leaving only the four other environmental variables as predictors for the dbRDA. These four constrained environmental variables were then transformed to the Euclidean distance to perform the RDA (i.e., dbRDA). Type II ANOVA was then used to test the significance of each environmental variable for explaining the genetic composition under 9999 permutations. We further analyzed the distribution of environmental variables along the ordination axes using the GLM. GLM analysis facilitates the elucidation of the importance of variables in the explanation of genetic composition under the dbRDA. We fit counters of current climate variables onto the ordination space of dbRDA plots to show the association of environmental variables with genetic divergence.

## Electronic supplementary material

Supplementary files
