# Supplementary material for: Continuation of the genetic divergence of ecological speciation by spatial environmental heterogeneity in island endemic plants
Source: Sci Rep. 2017 Jul 14;7:5465. doi: 10.1038/s41598-017-05900-1 (PMC5511155; doi:10.1038/s41598-017-05900-1)
Supplement: Supplementary file 1 — Supplementary files [file 41598_2017_5900_MOESM1_ESM.pdf]

Continuation of the genetic divergence of ecological speciation by spatial environmental heterogeneity in island endemic plants

Bing-Hong Huang, Chih-Wei Huang, Chia-Lung Huang, Pei-Chun Liao*

Department of Life Science, National Taiwan Normal University, Taipei 11677, Taiwan
Email addresses of authors:
Bing-Hong Huang: sohjiro4321@yahoo.com.tw
Chia-Lung Huang: biogun25@gmail.com

* Corresponding author: Dr. Pei-Chun Liao
Tel: +886-2-77346330
Fax: +886-2-29312904
Email: pcliao@ntnu.edu.tw

Table S1 Phenology of *Scutellaria playfairii* and *S. tashiroi* at each sampling site.

	Scutellaria playfairii		*Scutellaria tashiroi*		
Growth form	Stem erect, seldom tufted		Stem slender, procumbent, tufted		
Inflorescence	Terminal loose racemes		Axillary, rare terminal racemes		
Corolla color	Whitish-purple		Dark-purple		
Corolla form at base	Geniculate		Curved		
Population	Wutai	Wulu	Wulu	Lanyu	Taroko
Substrate	Shale	Shale	Shale	Agglomerate	Limestone
Soil	Sand	Loam	Sand	Clay	Sand
Longitude (E)	120.718137	121.047112	121.047111	121.510169	121.509524
Latitude (N)	22.739569	23.165561	23.165562	22.077472	24.180075
Altitude (m)	502	776	776	142	436
Current annual mean temp (℃)	19.4	16.8	16.8	23.9	17.2
Current annual precip (mm)	3658	2173	2173	2643	2338
LGM annual mean temp (℃)	14.1	14.3	14.3	20.6	13
LGM annual precip (mm)	3970	1920	1920	2353	2243

Table S2 Genetic diversity within populations for each microsatellite locus.

	Na		*Ne*		*I*		*Ho*		*He*		*F*	
	Mean	SE	Mean	SE	Mean	SE	Mean	SE	Mean	SE	Mean	SE
aus9-2*	1.000	0.000	1.000	0.000	0.000	0.000	0.000	0.000	0.000	0.000	NA	0.000
aus9-3*	1.000	0.000	1.000	0.000	0.000	0.000	0.000	0.000	0.000	0.000	NA	0.000
aus9-5*	1.000	0.000	1.000	0.000	0.000	0.000	0.000	0.000	0.000	0.000	NA	0.000
st5-179†	5.600	1.030	3.600	0.747	1.363	0.219	0.399	0.158	0.663	0.076	0.420	0.192
aus9-34	2.200	0.490	1.481	0.251	0.434	0.197	0.113	0.070	0.246	0.117	0.636	0.141
aus9-39	4.600	2.205	2.482	0.919	0.834	0.401	0.189	0.102	0.380	0.167	0.508	0.109
aus17-11	2.600	0.927	2.081	0.585	0.625	0.297	0.112	0.057	0.352	0.157	0.700	0.050
aus18-1	4.400	0.872	2.278	0.441	0.978	0.194	0.304	0.073	0.504	0.075	0.424	0.091
aus18-15	3.200	0.490	2.384	0.193	0.947	0.106	0.366	0.116	0.570	0.033	0.365	0.197
st4-47	1.800	0.800	1.630	0.630	0.299	0.299	0.074	0.074	0.152	0.152	0.515	0.156
st4-50	1.600	0.600	1.276	0.276	0.218	0.218	0.000	0.000	0.116	0.116	1.000	0.122
st5-48	1.600	0.400	1.386	0.239	0.309	0.193	0.020	0.020	0.196	0.120	0.903	0.061
st6-74	4.200	0.970	2.753	0.489	1.065	0.277	0.250	0.087	0.542	0.138	0.542	0.097
aus18-2	3.200	0.490	2.650	0.185	1.028	0.099	0.255	0.106	0.614	0.032	0.593	0.169
st10-8	1.800	0.374	1.212	0.104	0.261	0.111	0.040	0.040	0.152	0.069	0.814	0.144
st5-108	2.000	0.316	1.481	0.173	0.448	0.128	0.131	0.048	0.284	0.089	0.461	0.189
st5-6	1.800	0.800	1.435	0.435	0.275	0.275	0.100	0.100	0.137	0.137	0.270	0.144
st5-129	1.600	0.400	1.274	0.233	0.226	0.166	0.031	0.020	0.138	0.106	0.727	0.056
st5-165	2.200	0.583	1.652	0.412	0.468	0.228	0.175	0.113	0.273	0.130	0.452	0.142

* Positive-outlier locus
† Negative-outlier locus
Na, number of different alleles
Ne, number of effective alleles
I, Shannon's information index
Ho, observed heterozygosity
He, expected heterozygosity
F, fixation index

Table S3 Estimates of model parameters for the AM model through approximate Bayesian computation.

	$M_{pla \rightarrow tas}$	$M_{tas \rightarrow pla}$	N_{anc}	N_{pla}	N_{tas}	t_1	t_2	SMM	μ
Mode	0.100	0.100	3005	2719	1955	134474	111448	0.7789	0.0003
Mean	0.149	0.148	0.396	0.334	0.289	0.163	0.348	0.5473	0.2555
Median	1.067	1.062	6276	4977	4311	150300	280534	0.5637	0.0003
HPD (95% lower bound)	2.50E-5	2.38E-5	1000	1000	1000	1000	1009	0.1006	0.0002
HPD (95% upper bound)	3.768	3.768	16849	15608	13221	322325	839344	1.0000	0.0003

M, migration rate; *N*, effective population size; t_1, time of gene flow; t_2, divergent time; SMM, proportion inferred to fit to a sliding-mutation model; μ, mutation rate; HPD, highest posterior density

Table S4 General linear model (GLM) for testing the significance of five environmental variables along their transformed axis 1 of principal components (PC1).

	Coefficient	SE	t	Pr(>|t|)
Intercept	5.1520	0.5111	10.080	2.65E-11
alt	-0.0002	0.0001	-2.591	0.0145
AET	0.0026	0.0006	4.309	0.0002
bio3	-0.1493	0.0070	-21.473	<2E-16
bio13	-0.0042	0.0001	-33.898	<2E-16
bio14	0.0187	0.0008	22.797	<2E-16

Table S5 Summary results of distance-based redundancy analysis (dbRDA) for the eigenvectors of principal components (PC1 and PC2) of the genetic diversity. The formula of the dbRDA is SSR ~ alt + AET + bio3 + bio14.

	Inertia	Proportion	Rank
Total	1.01E+09		
Real total	1.01E+09	100.0%	
Constrained	9.16E+08	90.8%	2
Unconstrained	9.25E+07	9.2%	2
Imaginary	-3.72E-07		44

*Inertia is defined as the Euclidean distance squared.

Table S6 Significance tests for each constraint variable of the dbRDA on genetic diversity.

	df	Variance	F	Pr(>F)
alt	1	3.02E+08	248.159	0.0001
AET	1	3.17E+07	26.069	0.0001
bio3	1	7.63E+07	62.717	0.0001
bio14	1	5.06E+08	415.941	0.0001
Residual	76	9.24E+07		

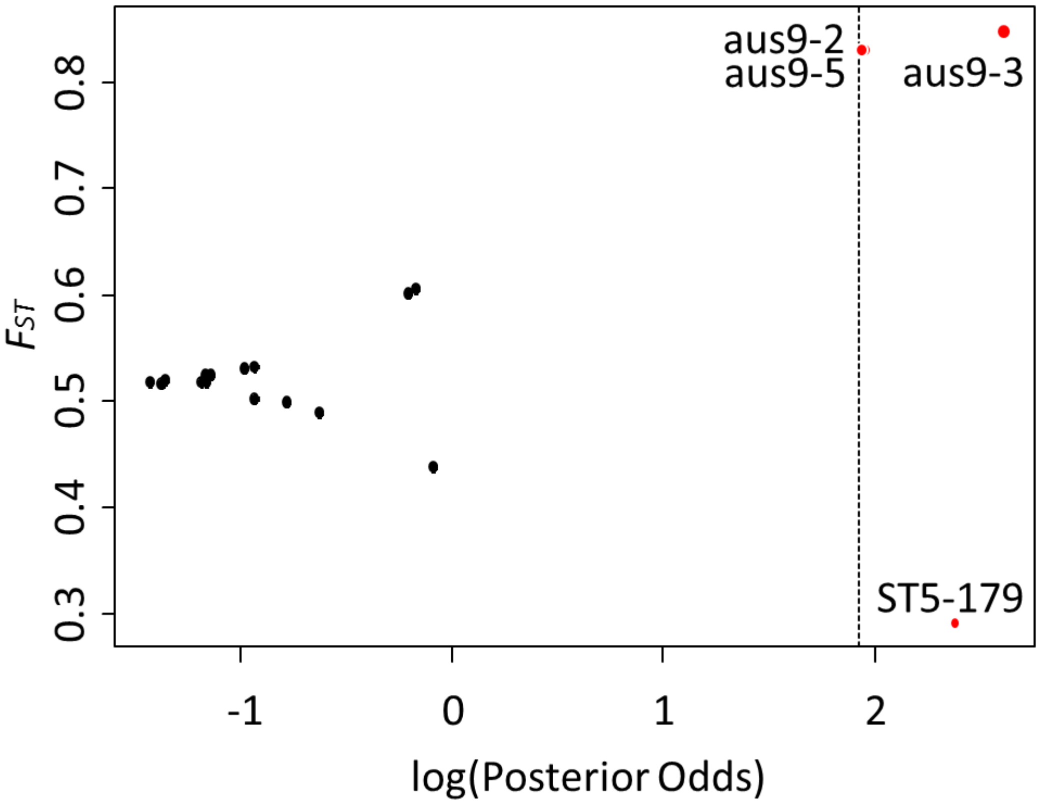

Figure S1 Plot of the BayeScan approach that finds three positive-outlier loci (aus9-2, aus9-3, aus9-5) and one negative-outlier locus (ST5-179) using a criterion of a Bayes Factor > 10. Note that aus9-2 and aus9-5 have same estimates of F_{ST}.

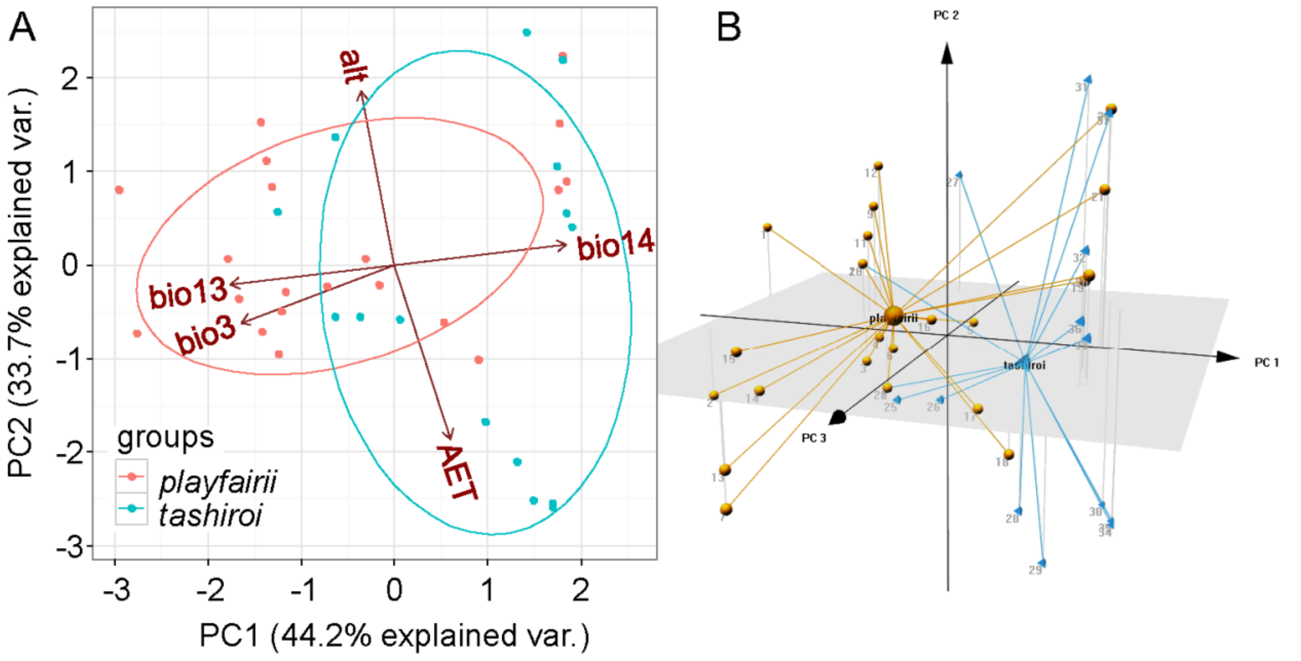

Figure S2 Principal component analysis (PCA) for the five environmental variables revealed in 2-dimensional (A) and 3-dimensional plots (B). The PCA result shows partial overlap of the niches of two *Scutellaria* species.

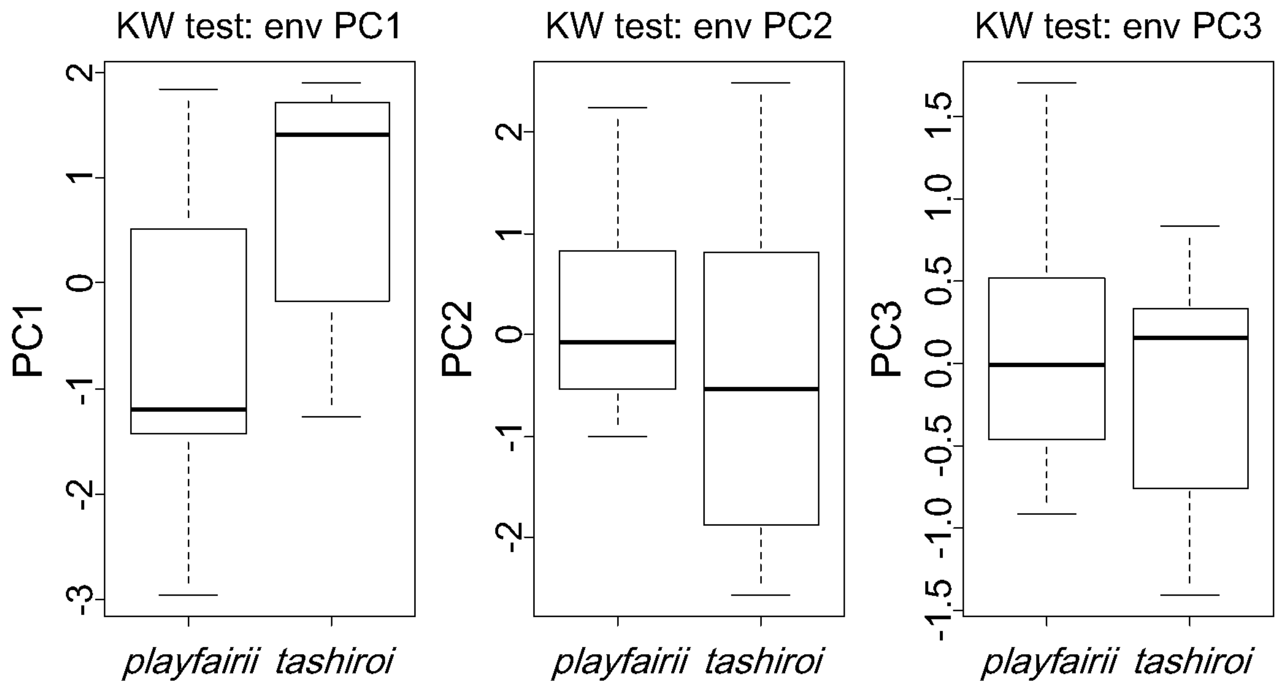

Figure S3 The Kruskal–Wallis test of the first three principal components of the environmental variables reveals significant niche divergence at PC1 between *S. playfairii* and *S. tashiroi*.

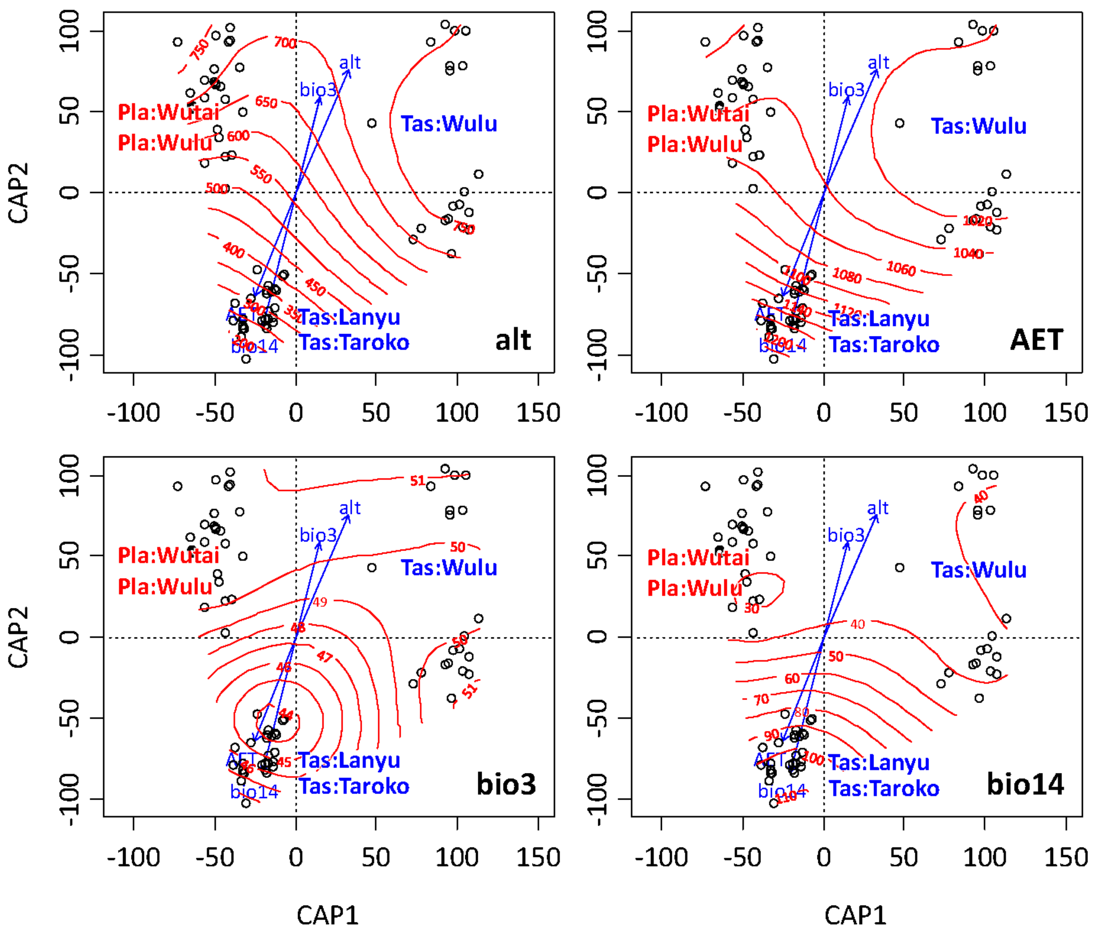

Figure S4 Scatter and ordisurf plots of the dbRDA for the four significant explanatory environmental variables.

Supplementary Note

Tests of correlation among genetic, geographic and environmental factors

The genetic distance was calculated by $F_{ST}/(1-F_{ST})$. The geographic distance was the great-circle distance, and the environmental distance was the Euclidean distance of five environmental variables (i.e., alt, AET, bio3, bio13, and bio14). To test the correlation between genetic and environmental distances, we also performed the partial Mantel test by conditioning on the geographic distance. The Mantel statistic was based on Spearman's rank correlation ρ with 9999 permutations. Associations among genetic, geographic, and environmental distances were evaluated by the Mantel test to determine if these three parameters are linearly correlated. A fit of the sampled populations to these parameters would suggest that the genetic composition of the sampled population is governed by geographic distances or local environmental differences. According to the Mantel tests among the genetic, geographic, and environmental distances, there were no significant correlations between each pair (geographic vs. environmental: ρ = 0.273; P = 0.225; genetic vs. geographic: ρ = -0.394; P =0.942; genetic vs. environmental: ρ = 0.006; P = 0.483). A further association test for environment and genetic distance is conditioning on the geographic distance by a partial Mantel test, which eliminates spatial effects on environmental differences. The partial Mantel also showed no significant correlation between genetic distance and environmental distance (ρ = 0.128; P = 0.383). These statistical values indicated that there are no linear correlations among the genetic, geographic, and environmental distances in our sampled populations. However, we have to remind that this analysis is calculated based on small number of populations (n = 5). The limited number of sampling may also reduce the power to detect the correlation between genetic and environmental factors.
